# Synthesis, Physical, Mechanical and Antibacterial Properties of Nanocomposites Based on Poly(vinyl alcohol)/Graphene Oxide–Silver Nanoparticles

**DOI:** 10.3390/polym12030723

**Published:** 2020-03-24

**Authors:** Mónica Cobos, Iker De-La-Pinta, Guillermo Quindós, María Jesús Fernández, María Dolores Fernández

**Affiliations:** 1 Department of Polymer Science and Technology, Faculty of Chemistry, University of the Basque Country (UPV/EHU), Paseo Manuel Lardizábal 3, 20018 San Sebastián, Spain; monica.cobos@ehu.es (M.C.); mjesus.fernandez@ehu.es (M.J.F.); 2 Department of Immunology, Microbiology and Parasitology, Faculty of Medicine and Nursing, University of the Basque Country (UPV/EHU), Barrio Sarriena s/n, 48940 Leioa, Spain; iker.delapinta@ehu.es (I.D.-L.-P.); guillermo.quindos@ehu.eus (G.Q.)

**Keywords:** poly(vinyl alcohol), graphene oxide, silver nanoparticles, nanocomposites, hybrids, antibacterial activity

## Abstract

The design of new materials with antimicrobial properties has emerged in response to the need for preventing and controlling the growth of pathogenic microorganisms without the use of antibiotics. In this study, partially reduced graphene oxide decorated with silver nanoparticles (GO–AgNPs) was incorporated as a reinforcing filler with antibacterial properties to poly(vinyl alcohol) (PVA) for preparation of poly(vinyl alcohol)/graphene oxide-silver nanoparticles nanocomposites (PVA/GO–AgNPs). AgNPs, spherical in shape and with an average size of 3.1 nm, were uniformly anchored on the partially reduced GO surface. PVA/GO–AgNPs nanocomposites showed exfoliated structures with improved thermal stability, tensile properties and water resistance compared to neat PVA. The glass transition and crystallization temperatures of the polymer matrix increased with the incorporation of the hybrid. The nanocomposites displayed antibacterial activity against *Staphylococcus aureus* and *Escherichia coli* in a filler content- and time-dependent manner. *S. aureus* showed higher susceptibility to PVA/GO–AgNPs films than *E. coli.* Inhibitory activity was higher when bacterial cells were in contact with nanocomposite films than when in contact with leachates coming out of the films. GO–AgNPs based PVA nanocomposites could find application as wound dressings for wound healing and infection prevention.

## 1. Introduction

Misuse and overuse of antibiotics has led to the emergence of multi-drug resistance microorganisms. To prevent and combat them it is important to develop new antimicrobial materials. In this context, antimicrobial polymers represent an alternative in order to avoid antibiotic resistance. An approach to prepare antimicrobial polymers involves the incorporation of an antimicrobial agent into polymer matrix during processing of the material. The combination of nanomaterials with polymers, in particular, metal nanoparticles and carbon-based nanomaterials, has been a method developed by several authors to prepare antimicrobial nanocomposites [1,2,3]. The excellent chemical, physical and biological properties exhibited by nanomaterials, attributed to their features such as size, shape, structure, high surface area, make them excellent candidates for the preparation of new materials. Silver nanoparticles (AgNPs) have been extensively used due to their outstanding antimicrobial activity against a broad range of microorganisms. Among carbon-based nanomaterials, graphene-based nanomaterials have also shown antimicrobial activity against a wide variety of pathogens [4,5,6,7,8,9,10]. Graphene oxide (GO), the oxidized form of graphene, has been reported to have antimicrobial properties, that are strongly affected by its physicochemical properties, such as sheet size, surface area, purity, structural defects, surface chemical properties, functional groups and degree of oxidation. In addition, the antibacterial activity is GO concentration, time and medium dependent [5]. Regarding AgNPs, the most important features influencing their antimicrobial activity are concentration, size and shape [11,12,13,14,15]. The poor colloidal stability of AgNPs in solutions leads to nanoparticle aggregation that reduces their surface area and increases their size, resulting in a weakening of the antimicrobial activity. The stabilization of AgNPs can be achieved by using GO as a substrate for supporting the nanoparticles. The oxygen functional groups on the surface of GO sheets act as sites for both nucleation and growth of nanoparticles, in addition stabilize AgNPs after growth. Antimicrobial properties of graphene oxide–AgNPs hybrids (GO–AgNPs) is improved compared to pristine AgNPs and GO, that has been attributed to the synergetic effect of AgNPs and GO [16,17]. AgNPs, GO and GO–AgNPs hybrids have been incorporated into polymers to produce polymer nanocomposites [18,19,20,21,22,23,24,25,26,27,28,29]. The resulting composites revealed antimicrobial properties. GO and AgNPs display photocatalytic activity that is enhanced when both are combined, due to the synergistic effect between them [30,31]. GO-AgNP hybrids can act as photocatalysts in both the environmental and industrial fields. Similar behavior has been reported by Yang et al. for Bi-based photocatalysts modified with Ag/AgCl [32]. Liu et al. have reported the preparation of composite membranes based on poly(vinyl alcohol)/poly(acrylic acid)/carboxyl-functionalized graphene oxide modified with silver nanoparticles with a remarkable photocatalytic capacity to degrade organic dyes [33]. The fabrication of cotton fiber coated by Ag/AgCl photocatalyst using polydopamine as adhesive agent was reported by Ding et al. [34]. These studies, however, made no analysis on the antimicrobial activity of those materials.

Polyvinyl alcohol (PVA), a synthetic polymer, has found applications in the biomedical area due to its properties such as biodegradability, biocompatibility, low toxicity, chemical and mechanical resistance, and good water solubility. However, it has some limitations such as the lack of antimicrobial properties, thus in biomedical applications biofilm formation can develop on its surface, acting as a potential source of persistent infections. PVA has been used as a suitable host material to synthesize polymer nanocomposites with different fillers, as the large amount of hydroxyl groups on its carbon backbone enables the formation of hydrogen bonding with hydrogen bond acceptor atoms contained in the reinforcing phase. Hydrogen-bonding interactions provides good dispersion of nanofillers, which is a crucial factor determining the polymer nanocomposite properties. GO reinforced PVA composites have been prepared and investigated by several authors [35,36,37,38,39,40]. These studies have reported improvement in properties at low filler content, being the interactions through hydrogen bonding between the hydroxyl groups of PVA and the abundant oxygenated functional groups on GO surface responsible for such enhancements. It has been reported that the incorporation of GO–AgNPs hybrids into PVA improved the antibacterial properties as compared to GO and AgNPs due to the synergistic effect between both nanomaterials [27]. PVA/GO–AgNPs composites can be prepared through two main routes, in situ and ex situ methods. The in situ approach is a one-step method that consists of chemical reduction of both metal precursor and GO sheets in the presence of the polymer. The ex situ method is based on the incorporation of previously synthesized GO–AgNPs hybrids into the polymer by melt compounding or solution blending.

In this study, we report the preparation of antibacterial PVA/GO–AgNPs nanocomposites by the ex situ approach and their characterization. Antibacterial GO–AgNPs hybrid, pre-synthesized by a one-step green approach using ascorbic acid as reducing agent of AgNO_3_ metal precursor, was mixed directly with PVA by solution blending to obtain PVA/GO–AgNPs films by casting. Physical, mechanical and antibacterial properties were investigated as a function of GO–AgNPs loading. The incorporation of GO–AgNPs hybrid with an AgNPs average size of 3.1 nm into PVA led to antibacterial composite films with improved thermal, mechanical and water resistance properties.

## 2. Materials and Methods

### 2.1. Materials

Graphite flakes were purchased from Alfa Aesar (Karlsruhe, Germany) (99.8%, 325 mesh), sodium nitrate (NaNO_3_) was obtained from Merck (Darmstadt, Germany), sulphuric acid (H_2_SO_4_, 98%), potassium permanganate (KMnO_4_), hydrogen peroxide (H_2_O_2_, 30 wt % aqueous solution), hydrochloric acid (HCl, 37% aqueous solution) and ammonium hydroxide were acquired from Panreac (Barcelona, Spain), while L-Ascorbic acid (L-AA), poly(vinyl alcohol) (PVA) (*M*_w_= 61,000 Da, degree of hydrolysis 98.0–98.8 mol %), silver nitrate (AgNO_3_) and phosphate-buffered saline (PBS) were supplied by Sigma–Aldrich (Munich, Germany, and St. Louis, MO, USA). *Escherichia coli* ATCC 25922 (Gram-negative bacteria) and *Staphylococcus aureus* ATCC 25923 (Gram-positive bacteria) were obtained from CECT (Spanish Type Culture Collection, Valencia, Spain), and brain heart infusion (BHI) broth, plate count agar (PCA) and Mueller–Hinton broth (MHB) were supplied by Condalab (Madrid, Spain). All chemicals were used as received without further purification. 

### 2.2. Synthesis of Graphene oxide (GO) and Graphene oxide–Silver Nanoparticles Hybrid (GO–AgNPs Hybrid)

Graphene oxide (GO) was synthesized from natural graphite flake following a modified Hummers method as previously described [41]. Details of the synthesis are described in the Supplementary Materials (Preparation of Graphene Oxide (GO)). We used the in situ approach for the synthesis of GO–AgNPs hybrid, which consists of simultaneous reduction of metal salt and GO using L-AA as a green reductant as described in our previous work [42]. Briefly, an aqueous dispersion of graphite oxide (GrO) (0.5 mg/mL) was sonicated for 1 h to obtain GO. Then, after adjusting the pH to 10 by adding ammonium hydroxide, a silver nitrate solution was added (to reach 1.5 mM) under stirring and in the absence of light, and the mixture was heated to 60 °C in an oil bath. Subsequently a L-AA solution was added at a concentration which gave a weight ratio of L-AA to silver nitrate of 2.07. The reaction mixture was cooled, subjected to dialysis, and centrifuged after being at 60 °C for one hour. The obtained product was freeze dried to obtain GO–AgNPs powder. 

### 2.3. Preparation of PVA and PVA/GO–AgNPs Nanocomposite Films

A solvent casting method was used to prepare neat PVA and composite films. An aqueous solution of PVA was prepared by dissolving 5 wt % PVA in deionized water at 100 °C under constant stirring for one hour. The solution was cast on a plastic petri dish and left to dry at room temperature before the neat PVA film was peeled off. PVA/GO–AgNPs composites were prepared by mixing an aqueous GO–AgNPs dispersion previously sonicated with an aqueous solution containing 5 wt % PVA under stirring for 1 h, and sonication for another 1h. The mixture was then cast as film at room temperature. The as-prepared films were heated at 60 °C under vacuum for three days before use. The samples were labelled as PVA/GO–AgNPs*x*, were x denotes the wt % of the filler.

### 2.4. Physicochemical Characterization and Mechanical Properties

FTIR spectroscopy (Thermo Nicolet iS10, Thermo Fisher Scientific, Madison, WI, USA), Ultraviolet–visible absorption spectroscopy (UV-vis) (Perkin Elmer Lambda 25, Shelton, CT, USA), X-ray photoelectron spectroscopy (XPS) (SPECS Phoibos 150 1D-DLD, Berlin, Germany), Raman spectroscopy (Renishaw Invia, Gloucestershire, UK), X-ray diffraction (XRD) (Malvern Panalytical X’PERT PRO, Almelo, Netherlands), Scanning electron microscopy (SEM) (Hitachi S-4800, Tokyo, Japan) and Transmission electron microscopy (TEM) (Philips Tecnai G2 20 TWIN, Eindhoven, Netherlands) were employed to evaluate the structure and the morphology of the samples. Thermogravimetric analysis (TGA) (TA-TG-Q-500, New Castle, DE, USA) and differential scanning calorimetry (DSC) (Mettler Toledo DSC 3+, Greifensee, Switzerland) were used for the thermal characterization, and tensile tests (Instron 5967, Norwood, MA, USA) to determine the mechanical properties. Protocols of the characterization are shown in the Supplementary Materials (Physicochemical Characterization).

### 2.5. Water Absorption

The water absorption of PVA/GO–AgNPs nanocomposite films was estimated by immersing the specimens in deionized water at 25 °C for 24 h. Prior to immersion, the samples were dried at 60 °C for three days to a constant weight, then placed in a desiccator to cool, and weighed (*W*_0_). The films were then immersed in deionized water at 25 °C. After 24 h, the specimens were removed, the excess water on the surface was wiped with a filter paper and the films were weighed (*W*_w_). Then, after drying the samples under vacuum for three days at 60 °C, they were weighed (*W*_d_). Three replicates per sample were tested. The following Equation (1) was used to calculate the total water content (*W*_t_) for each sample:

(1) $$W_{t}\left(\%\right)=\frac{W_{w}-W_{d}}{W_{0}}\times 100$$

### 2.6. Microbial Strains and Culture

Gram-negative bacterium *Escherichia coli* ATCC 25922 and Gram-positive bacterium *Staphylococcus aureus* ATCC 25923 were evaluated. Briefly, bacteria were cultured on PCA for 24 h at 37 °C, and inoculum was prepared from single colonies grown to stationary phase in BHI broth at 37 °C overnight in an orbital incubator under 100 rpm. Broths were centrifuged (3000× *g*, 10 min) and washed twice with PBS. A cell suspension adjusted to a cell density equivalent to 0.5 McFarland (representing approximately 1–5 × 10^8^ cells/mL) was prepared using sterile saline. Viable counts were enumerated after overnight incubation at 37 °C onto PCA.

### 2.7. Antibacterial Activity Assay

A modification of the EUCAST dilution method was used to assess the antibacterial activity [43]. The antibacterial activities of both nanocomposite films in direct contact with bacterial cells and the leaching from the films after their immersion in PBS at 37 °C for 24 h were evaluated.

The antibacterial activity was tested in 96-well microtiter plates in a volume of 100 µL of MHB at 2-fold the desired final concentration. The inoculum was diluted 1:10 with sterile water and each well was inoculated with 100 µL to give a final concentration of 5 × 10^6^ CFU/mL. Nanocomposite film discs of 0.6 cm in diameter sterilized under ultraviolet light were placed in the wells of the plate in triplicate for each material and for each day. The plates were then incubated at 37 °C, and every 24 h the samples were removed with sterile clamps until 72 h. The absorbance of the culture monitored with a microplate reader (iMark, BioRad, Hercules, CA, USA) at 450 nm, and the quantification of colony-forming units (CFU) present in the wells after dilution and seeding on PCA were used to determine bacterial growth in each well. This experiment was repeated three times during different weeks.

On the other hand, an antibacterial activity assay was performed to determine whether the lixiviates from PVA/GO–AgNPs composite films showed a growth inhibitory effect against *E. coli* and *S. aureus*. The test was carried out in 100-well microtiter plates at 37 °C (Labsystem, Helsinki, Finland). The nanocomposite films were cut out to a size of 0.6 cm in diameter, sterilized under ultraviolet light, placed in the wells of the plates in triplicate for each material and 100 µL of PBS were added to each well. Plates were incubated for 24 h at 37 °C. Then nanocomposite films were removed aseptically with a clamp and other 100 µL of inoculum were added. The microtiter plates were then placed in a microplate reader (Bioscreen C, Labsystem, Helsinki, Finland), which was set up to measure the bacterial growth by reading the absorbance at 450 nm every hour for 72 h at 37 °C. The experiment was performed in triplicate and differences between PVA (used as control with no antibacterial activity) and PVA/GO–AgNPs nanocomposite films were evaluated by analysis of variance (ANOVA) as explained below. 

### 2.8. Data Analysis – Statistical Analysis

Data were analyzed using analysis of variance (ANOVA) followed by Tukey or Games-Howell’s correction depending on the homogeneity of variances (tested by Levene test) and significant differences between neat PVA and nanocomposites were determined (*p* value < 0.05). Calculations were performed with statistical software SPSS 24 (IBM SPSS Statistic, New York, NY, USA). 

## 3. Results and Discussion

Results and discussion of characterization of GO and GO–AgNPs hybrid are provided in the Supplementary Materials (Figures S1–S4). Table 1 shows the reaction conditions for GO–AgNPs hybrid and the average size of the AgNPs anchored on the GO surface as determined by TEM. 

### 3.1. Characterization of PVA/GO–AgNPs Nanocomposites

#### 3.1.1. X-Ray Diffraction Analysis

**Figure 1:**
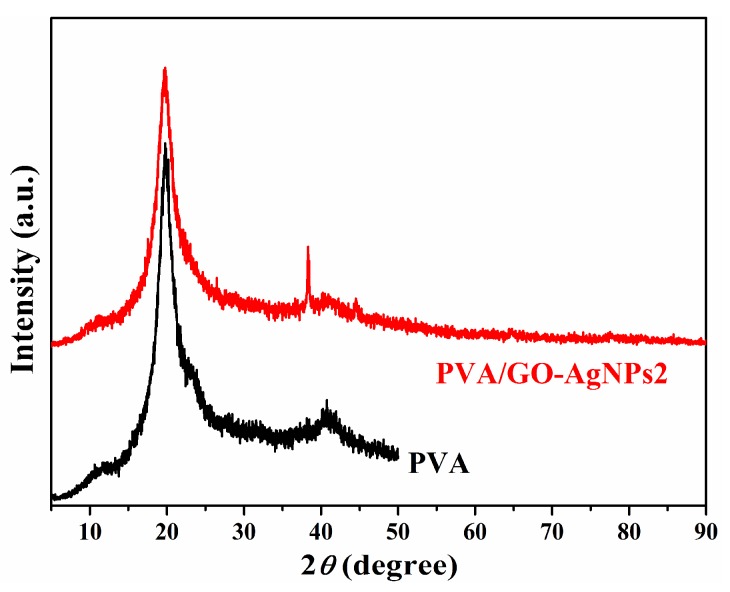
XRD patterns of neat poly(vinyl alcohol) (PVA) and PVA/GO–AgNPs2 nanocomposite.

PVA/GO–AgNPs composites containing 0.5, 1, 2 and 5 wt % GO–AgNPs hybrid were prepared. Figure 1 shows X-ray diffraction pattern of neat PVA and PVA/GO–AgNPs composites with 2 wt % of GO–AgNPs in casted film form. The pattern of neat PVA film displays two peaks at 2θ = 19.8° and 40.8°, while that of the composite film shows two additional peaks at 2θ = 38.3° and 44.2°, corresponding to the (111) and (200) crystal planes of the face-centred cubic crystal structure of AgNPs, respectively.

#### 3.1.2. Microstructural and Morphological Characterization: SEM and TEM Analysis

**Figure 2:**
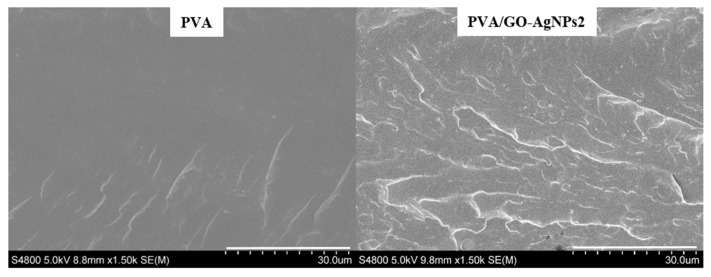
SEM images of the fracture surfaces of neat PVA and PVA/GO–AgNPs2 nanocomposite.

**Figure 3:**
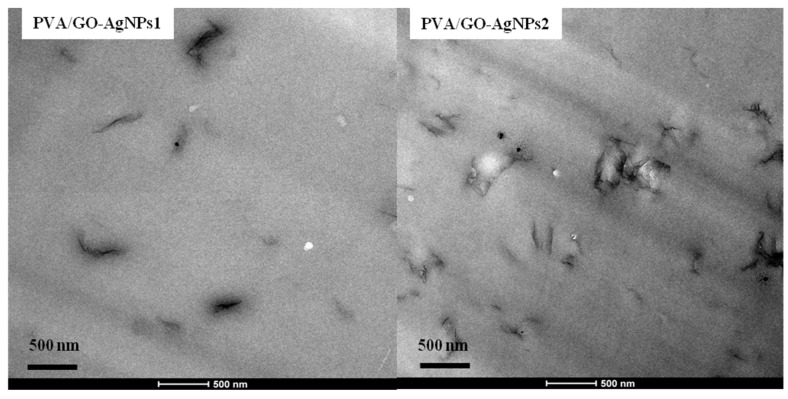
TEM images of PVA/GO–AgNPs nanocomposite films with 1 wt % and 2 wt % GO–AgNPs.

Morphological features of PVA/GO–AgNPs composites was analysed by SEM and TEM. Figure 2 illustrates SEM micrographs for the fracture surfaces of neat PVA and PVA/GO-AgNPs2 nanocomposite. The neat PVA fracture surface shows a smooth surface. However, the fracture surface morphology is different after the GO–AgNPs hybrid is incorporated. A rougher surface, with a layered structure is observed in the case of the PVA/GO–AgNPs2 nanocomposite film. The composite sample shows no agglomerates, indicating that the GO–AgNPs hybrid disperses well in the PVA matrix and that a strong interfacial interaction exists between the hybrid and the matrix. Figure 3 shows TEM images of PVA/GO–AgNPs nanocomposite films filled with 1 wt % and 2 wt % GO–AgNPs. The images reveal the good dispersion obtained, where exfoliation of most of the GO sheets is seen. 

### 3.2. Thermal Analysis of PVA/GO–AgNs Nanocomposites

#### 3.2.1. Thermal Properties

**Figure 4:**
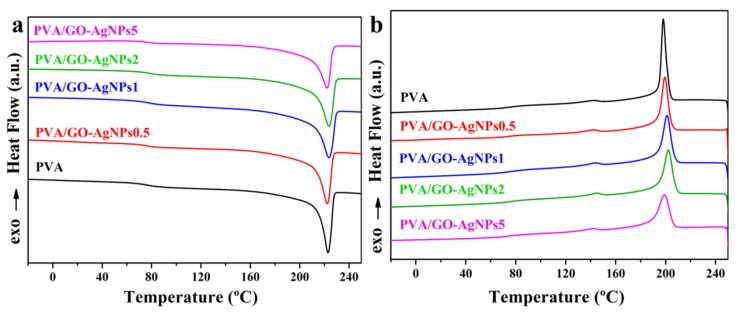
DSC curves of neat PVA and PVA/GO–AgNPs composites. (**a**) Second heating scan; (**b**) first cooling scan.

Thermal transition behavior was investigated by DSC. Thermal transition temperatures and enthalpies of neat PVA and PVA/GO–AgNPs nanocomposites are reported in Table 2. The crystallization temperature (*T_c_*) and crystallization enthalpy (Δ*H*_c_) were estimated during the first cooling scan, whereas the glass transition temperature (*T*_g_), melting temperature (*T*_m_) and melting enthalpy (Δ*H*_m_) during the second heating scan. The degree of crystallinity (*X_c_*) of PVA was calculated from the second heating scan by using the following equation:

(2) $$X_{c}\left(\%\right)=\left[\frac{∆H_{m}}{∆H_{m}^{0}\left(1-\emptyset \right)}\right]\times 100$$

where Δ*H*_m_ is the enthalpy of fusion of the PVA and PVA/GO–AgNPs nanocomposites, Δ*H^0^*_m_ is the enthalpy of fusion of the 100% crystalline PVA (141.932 J/g) [44], and *ϕ* is the weight fraction of filler in the composite. The crystallinity of nanocomposites remained constant with the incorporation of GO–AgNPs into PVA. Figure 4 shows the corresponding thermograms. 

The effect of GO–AgNPs hybrid addition to PVA on the thermal transitions of the PVA matrix can be seen in the data displayed in Table 2. The addition of GO–AgNPs hybrid to the PVA matrix increases the *T_g_*, and the higher the hybrid content, the higher the *T_g_* value (Table 2). Neat PVA displays a *T_g_* of 76.5 °C, while for PVA/GO–AgNPs5 composite film, the *T_g_* rises to 83.0 °C, with an increment of 6.5 °C. The enhanced *T_g_* can be explained by the reduced polymer chain mobility due to the interfacial interactions between the hybrid and the PVA. The incorporation of GO–AgNPs hybrid also resulted in a slight increase in crystallization temperature (*T*_c_), which can be due to the nucleating effect of GO–AgNPs on the PVA crystallization. However, the melting peak temperature and enthalpy of PVA remained unchanged by the addition of the hybrid. The crystallization enthalpy of the nanocomposites and the degree of crystallinity (*X_c_*) of PVA were slightly reduced upon increasing the content of the hybrid.

#### 3.2.2. Thermal Stability

**Figure 5:**
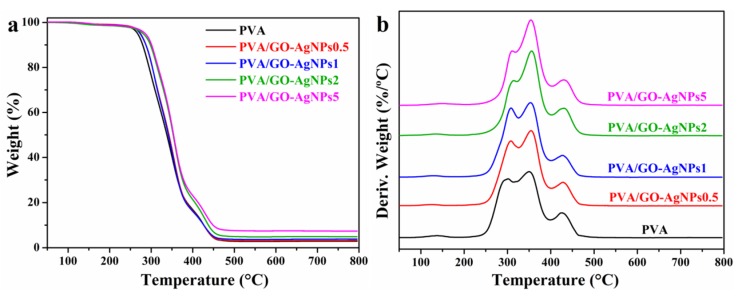
(**a**) TG and (**b**) DTG curves of neat PVA and PVA/GO–AgNPs nanocomposites in nitrogen.

The influence of GO–AgNPs hybrid on the thermal stability of PVA was evaluated by TGA. TG and DTG curves are displayed in Figure 5, and Table 3 summarizes the data collected from these curves. Two stages are observed in the thermal decomposition of PVA, which occur in the following temperature regions: the first one in the range between 200 and 400 °C, and the second one between 400 and 500 °C. It is during the first step when the greatest weight loss occurs (more than 80%), which corresponds to the dehydration, chain scission and decomposition of polymer backbone [45]. About 3% of the initial weight remained at 800 °C as carbon residue. From the DTG curve of the PVA two superimposed processes can be observed during the first decomposition phase (Figure 5b), with peaks appearing at 302 and 350 °C. The intensity of both peaks is quite similar. PVA/GO–AgNPs samples show a decomposition pattern similar to that shown by neat PVA. The DTG curves, however, show that the intensity of the peak at around 355 °C increases with respect to that of the peak at around 310 °C as the content of the hybrid increases beyond 1 wt %. The second peak shifts to a higher temperature, between 6 and 12 °C. 

Thermal decomposition temperatures for 5 (*T*_5%_ ) and 50% (*T*_50%_) weight loss increase after incorporation of the GO–AgNPs hybrid, the more significant increase being with 2 and 5 wt % GO-AgNPs, 9–15 °C and 14–16 °C, respectively. Therefore, the addition of GO–AgNPs hybrid improves the thermal stability of PVA, which can be due to the high thermal stability of the partially reduced GO that results from the synthesis of the hybrid. GO–AgNPs filler acts as a retardant agent for PVA decomposition. Char residue at 800 °C is higher for composites than for neat PVA. The higher the filler content, the higher the residue.

### 3.3. Mechanical Properties of PVA/GO–AgNPs Nanocomposites

**Figure 6:**
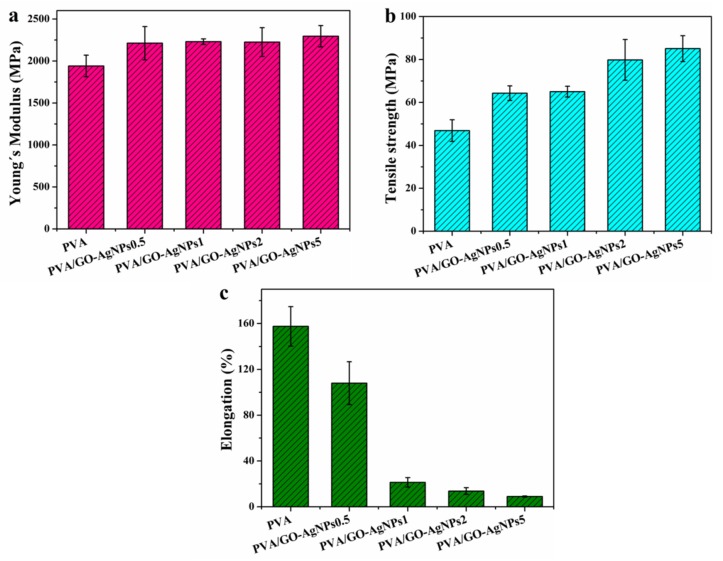
Tensile properties of neat PVA, and PVA/GO–AgNPs nanocomposites. (**a**) Young´s modulus; (**b**) tensile strength; (**c**) elongation at break.

It has been demonstrated the enhancement of mechanical properties of polymers by incorporating graphenic materials [46,47]. Tensile tests were performed to determine the mechanical properties of polymer matrix, being the elastic modulus (Young´s modulus), tensile strength at break and elongation at break the measured properties. The effect of GO–AgNPs hybrid content on the mechanical properties of PVA is shown in Figure 6. For 0.5 wt % GO–AgNPs incorporation, statistically significant differences in the elastic modulus and tensile strength at break of PVA were observed (*p* < 0.05). The increase of modulus was about 13%, while that of tensile strength was 38%. A further increase of GO–AgNPs content up to 5% led to an increment of around 18% and 80% in elastic modulus and tensile strength, respectively, when compared with neat PVA (*p* < 0.001). A severe and gradually decrease in the elongation at break is observed after the addition of GO–AgNPs hybrid, being the most important drop with 5 wt % GO–AgNPs, about 94 %, as compared with neat PVA (*p* < 0.001). The enhanced mechanical properties is attributed to the superior mechanical properties of GO [48] and the reinforcement effect of AgNPs. The constrained polymer chain mobility due to the interfacial interaction between PVA and GO–AgNPs hybrid leads to highly brittle PVA/GO–AgNPs composites.

Yang et al. developed PVA/GO nanocomposites using the solution casting method with GO loadings up to 3.5 wt % [38]. Both the tensile strength and elastic modulus were improved, while the elongation at break was significantly reduced. Zhao et al. [36] and Yang et al. [49] prepared PVA/graphene composites through a GO/PVA solution reduction process using hydrazine. Elastic modulus and tensile strength increased with rising graphene content, and the elongation at break decreased. Improved mechanical properties of PVA/GO prepared by solution mixing and PVA/graphene nanocomposites developed by chemical reduction of GO in the presence of polymer at low GO loadings were reported by Bao et al [39]. Manna et al. reported the improvement of mechanical properties of poly(vinylidene fluoride) when filled with AgNPs [50]. Mbhele et al. studied the mechanical properties of AgNPs-filled PVA and observed that its incorporation increased the elastic modulus and the stress at break of PVA [51]. In our previous work, AgNPs–GO-loaded PVA exhibited better mechanical properties than GO-loaded PVA [25]. Liu et al. studied the effect of GO–AgNPs hybrids on the tensile mechanical behavior of PLA. The authors observed that PLA/GO–AgNPs composites had higher tensile modulus and tensile strength than PLA/GO [29]. 

### 3.4. Water Absorption of PVA/GO–AgNPs Nanocomposites

**Figure 7:**
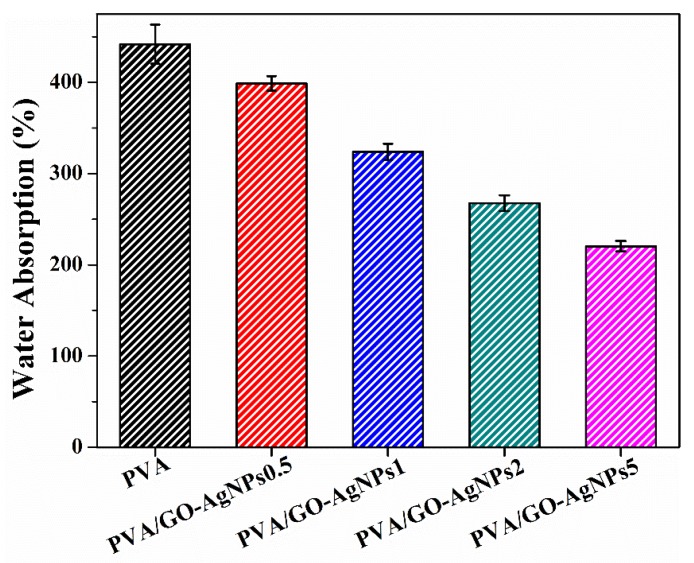
Water absorption of neat PVA and PVA/ GO–AgNPs nanocomposites after 24 h immersed in deionized water at room temperature.

Previous studies have revealed that graphene and GO decorated with AgNPs based PVA nanocomposites show a considerable improvement in water resistance compared to the neat polymer matrix [25,52,53]. The water uptake of pure PVA and PVA/GO–AgNPs nanocomposite films is displayed in Figure 7. The presence of GO–AgNPs results in a lower absorbed water content. A higher GO–AgNPs content leads to a lower amount of water uptake. A reduction of absorbed water between 10% and 50% is observed after the incorporation of GO–AgNPs into PVA. The hydrophilic character of PVA is responsible for the high-water absorption of this polymer. Water resistance depends on the interactions between water and polymer chains. The stronger the interfacial adhesion between the PVA and the hybrid, the less hydrophilic groups can interact with water. Therefore, the enhancement of water resistance can be attributed to interactions between the polymer and the hybrid through strong hydrogen bonds. In addition, a constrained polymer region is generated resulting from hydrogen bonding that prevents the water absorption [54,55].

### 3.5. Inhibition of Bacterial Growth by PVA/GO–AgNPs Nanocomposites

**Figure 8:**
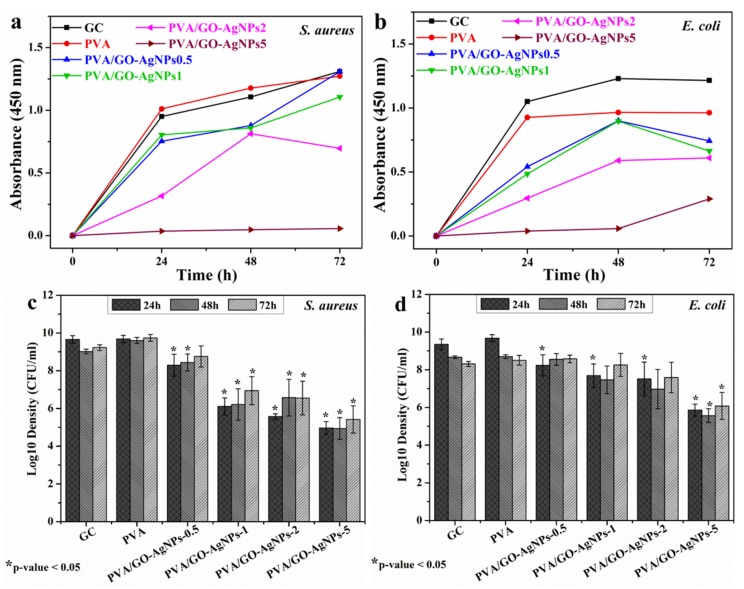
Absorbance of (**a**) *S. aureus* growth curves and (**b**) *E. coli* growth curves after different times of exposure to PVA/GO–AgNPs composite films. Viable counts of (**c**) *S. aureus* and (**d**) *E. coli*.

A wide range of bacteria, viruses, and fungi are responsible for healthcare-associated infections (HAI). Among bacteria, *E. coli* and *S. aureus* are the most common involved in severe HAIs. Many nosocomial strains of these bacteria are resistant to currently used antibiotics, causing major therapeutic failures, complicating the therapy of HAIs and prolonging the hospital stay of patients. Gram-negative strain *E. coli* ATCC 25922 and Gram-positive strain *S. aureus* ATCC 25923 were used as bacteria models to evaluate the antibacterial activity of PVA/GO–AgNPs composite films. The cell growth inhibition of the selected bacterial strains was estimated by the two following methods: measurement of culture absorbance and CFU counting. Figure 8 displays the effect of PVA/GO–AgNPs composite films with different GO–AgNPs contents on the growth of *S. aureus* and *E. coli* cells. As seen from Figure 8, the antibacterial properties of the PVA/GO-AgNPs composite films are time and GO–AgNPs loading dependent. On the basis of the absorbance measurements, nanocomposite samples with 0.5 and 1 wt % GO–AgNPs displayed no inhibition of *S. aureus* cells over the time investigated. By increasing the GO–AgNPs content up to 2 wt % a reduction in bacterial growth is observed only after 24 h of exposure, while 5 wt % GO-AgNPs has a remarkable effect on cell growth, since a complete inhibition effect is achieved after 24 h of exposure, which is maintained by the end of the period investigated (Figure 8a). The growth of *E. coli* cells loaded with PVA/GO–AgNPs composite films is shown in Figure 8b. The incorporation of 0.5 and 1 wt % GO-AgNPs into the PVA matrix exhibits a moderate reduction in *E. coli* bacterial growth upon 24 h of exposure, beyond that time not inhibitory effect is observed. In the case of PVA/GO–AgNPs2 composite film, inhibition of cell growth can be observed after 24 h of exposure, beyond that time the inhibitory effect is reduced, although it is greater than for PVA films containing 0.5 and 1 wt % GO–AgNPs. The growth of *E. coli* cell population is completely suppressed after 24 and 48 h of exposure in the presence of PVA films filled with 5 wt % GO-AgNPs. After 72 h this sample shows reduced antibacterial activity. On the other hand, as it can be seen from Figure 8c,d, the growth of both *S. aureus* and *E. coli* cell populations is reduced in the presence of PVA film filled with 0.5 to 5 wt % GO–AgNPs, and the composite sample film with the highest GO–AgNPs content exhibits the strongest bacterial growth inhibitory efficiency. 

By comparing the effect of PVA/GO–AgNPs nanocomposite films on both strains, it can be seen that *E. coli* strain is more resistant than that *S. aureus* to nanocomposite films with any GO–AgNPs hybrid content. In addition, the presence of GO–AgNPs hybrid in PVA/GO–AgNPs films leads to a longer-term antibacterial efficacy against *S. aureus* than against *E. coli*. These findings are in accordance with results previously reported by us [25] and by other authors [24,28]. Gram-negative and Gram-positive bacteria have two structurally different types of cell wall. The cell wall of the Gram-positive bacteria is a thick wall containing many peptidoglycan layers. However, Gram-negative bacteria have two membranes, with structures and compositions that are different. The inner one is the cytoplasmic cell membrane, the outer one has as a main constituent lipopolysaccharide (LPS), and between them, there is a thin peptidoglycan layer. LPS may act as a protective barrier preventing the penetration of toxic compounds into Gram-negative bacterial cells. 

**Figure 9:**
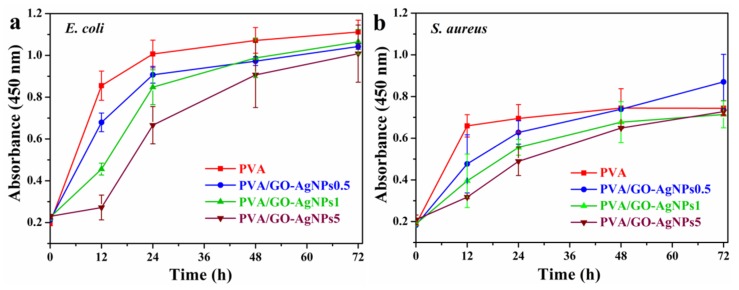
Absorbance of (**a**) *E. coli* growth curves and (**b**) *S. aureus* growth curves after different times of exposure to the leaches from PVA/GO–AgNPs composite films (for 24 h at 37 °C in PBS).

Leaching of antimicrobial agents from PVA/GO–AgNPs composite films after immersion in PBS (for 24 h at 37 °C) may exert a bacterial growth inhibitory effect. AgNPs and Ag^+^ ions are the antimicrobial agents that could leach out of the composite films. The evaluation of the antimicrobial effect of the leaches was estimated by measuring the absorbance at 450 nm (Figure 9). PVA film was used as growth control since it does not release any antimicrobial agent. The lixiviates from all PVA films containing GO–AgNPs showed significant bacterial growth inhibition compared to neat PVA in the first 12 h when they were in contact with bacteria. PVA/GO–AgNPs5 film displayed statistically the highest inhibitory activity against *E. coli* (*p* < 0.01), and after 24 h of exposure, the PVA/GO–AgNPs5 film was the only one that exhibited growth inhibition (*p* < 0.01) (Figure 9a). As for *S. aureus*, PVA/GO–AgNPs0.5 showed no inhibitory effect on this bacterium growth over the period of time studied, and both PVA/GO–AgNPs1 and PVA/GO–AgNPs5 did show it up to 24 h exposure (*p* < 0.05), although there were no significant differences between them (Figure 9b). After 48 h, none of the materials tested showed statistically significant differences with both microorganisms (*p* > 0.05) when compared with PVA.

In order to assess the possible release of silver nanoparticles from the polymer matrix, leaching tests were performed. For each PVA/GO-AgNPs sample, specimens with dimensions of 2 × 2 cm^2^ were immersed in 10 mL deionized water at 25 °C for 24 h. The solutions were then analyzed by UV-vis spectroscopy for the detection of the surface plasmon resonance absorption band of silver nanoparticles, but in none of the samples was the SPR band observed (Figure S5 of the Supplementary Materials). This indicates that there was no AgNPs in solution, suggesting that silver nanoparticles are firmly attached to GO, immobilized, and that a strong interaction has been established between GO–AgNPs hybrid and polymer matrix. Thus, the stability of the hybrid is confirmed. Therefore, it can be inferred that the antibacterial activity observed in the leachates released from the films is due to the leaching of silver ions (Ag^+^).

It has been shown that GO has antibacterial activity against Gram-positive and Gram-negative bacteria, but its mechanism of antibacterial action remains under debate. Physical and chemical interactions have been proposed to be responsible of its antibacterial activity [4,56]. The direct contact interaction of the sharp edges of graphene oxide sheets with the bacteria has been found to result in damage to the bacterial cell membrane. The interaction of GO sheets with bacteria by mechanically trapping or wrapping, which isolates them from the environment leading to cell dead, has also been suggested [6,7,57]. Other studies have claimed that oxidative stress is the major contributor to the antibacterial action of GO [5,56]. 

Although the mode of antibacterial activity of AgNPs is not fully understood yet, it has been recognized that the antibacterial mechanism of AgNPs depends on nanoparticle-cell interactions and/or silver ions interactions. Some authors suggest that the toxic effect is due to particle-only effects [58,59], others, however, attributed it to Ag^+^ ions alone [60], whereas there are others that claim that both nanoparticles and ions contribute to the toxicity [61,62,63,64,65,66]. In the former case, nanoparticles attack bacteria through direct contact with cell wall, since due to their high surface/volume ratio AgNPs can effectively contact microorganisms. After contacting, changes in membrane morphology have been observed, which leads to an increase in permeability, allowing nanoparticles penetration into cell membrane that affects the transport activity through the plasma membrane [67]. As a result, various vital cell functions are obstructed, leading to cell death. Sondi et al. [68], Morones et al. [58] and Agnihotri et al. [69] confirmed the incorporation of AgNPs into the *E. coli* membrane by electron microscopy. Other researchers, however, have claimed that the toxicity of AgNPs is attributed exclusively to the Ag^+^ ions released from the oxidized AgNPs surface in aerobic conditions [60]. Ag^+^ ions interact with thiol-containing proteins in the cell wall and affect their functions. The properties of nanoparticles such as size and shape indirectly influence the toxicity of AgNPs. The higher the specific surface area, the faster is the rate of silver particle dissolution. Small AgNPs release more Ag^+^ ions than the large ones, an according to the study by Sotiriou et al. the antibacterial performance of AgNPs with an average size <10 nm is governed by the Ag^+^ ions released from their surface [70]. Finally, there are some researchers that have stated that the toxic action of AgNPs is determined by a synergistic antibacterial effect between the AgNPs and the Ag^+^ ions released [61,69]. 

After 24 h of incubation, the results of the antibacterial experiments (Figure 8a,b and Figure 9) show that the inhibitory effect was higher when bacterial cells were in direct contact with PVA/GO–AgNPs composite films than when they were exposed to leaching of silver ions from PVA/GO–AgNPs composite films. From this result, it can be inferred that both the cell direct contact and penetration of the nanoparticles, together with the released silver ions from the oxidized surface of AgNPs, contribute to the toxic activity. In a previous work we found that the incorporation of 2 wt % GO to PVA resulted in a composite that lacked antibacterial activity against *E. coli* and *S. aureus* [25]. Therefore, we can conclude that AgNPs may be considered the main contributors to the bactericidal effect to PVA. In PVA/GO–AgNPs nanocomposite films GO–AgNPs hybrid can be emerging on the surface and embedded inside. GO–AgNPs sheets exposed at the surface of the film can have a direct contact with the bacteria, while those present in the bulk of the polymer cannot. The only possible mechanism of the antibacterial action of the latter is through the release of Ag^+^ ions. The water from the bacteria medium containing dissolved oxygen diffuses into the polymer and reaches the AgNPs embedded in the polymer, allowing their oxidation, and generating Ag^+^ ions that diffuse to the surface of the film and attack the adsorbed bacteria.

PVA has been used in biomedical and pharmaceutical applications as mentioned previously. Graphene derivatives and graphene-inorganic hybrid materials are also used as biomedical materials due to their unique properties [71]. Biomedical applications of these nanomaterials include drug and gene delivery, biosensing and bioimaging systems, tissue engineering and other therapeutic applications. The cytotoxicity of nanomaterials is of particular concern when it comes to biomedical applications. The interaction of AgNPs with cells is strongly influenced by their size, shape, surface coatings and aggregation [72]. Studies reported in the literature have shown that the specific physicochemical characteristics of graphenic materials, such as surface area, layer number, lateral dimension, surface chemistry and impurities contribute to the toxicity [73]. Moreover, the cytotoxicity of these nanomaterials is affected by the cell type, dose and exposure time. Cytotoxicity increases with the decrease of AgNPs size while the same concentration is maintained [74,75]. Likewise, an increase in dose leads to an increase in cytotoxicity. On the other hand, silver nanoparticles embedded in polymer matrices show less cytotoxicity than bared ones. Sowa-Söhle et al. [76] found that thermoplastic polyurethane-silver nanoparticle composites with nanosilver concentrations from 0.01 to 1.0 wt % were non-cytotoxic to mouse fibroblass cells but toxic to bacteria. Oliveira et al. [77] reported the preparation of PVA-Ag hydrogel samples with 0.25 and 0.50 wt % silver precursor. The results revealed the non-cytotoxicity of the materials to mouse fibroblast cells and antimicrobial activity towards bacteria and fungi. The concentration of nanoparticles necessary to induce toxicity in human cells is much higher than that needed to exert antimicrobial activity [78,79]. Regarding the cytotoxicity of graphene nanomaterials, it has been demonstrated the reduced cytotoxicity of graphenic materials when incorporated into polymer matrices [28,80]. Ma et al. found that the cytotoxicity of PVA/graphene nanocomposite fibers to human cells was low and graphene content dependent [28]. Based on the above-mentioned analysis, and according to the improved physical and antibacterial properties, the developed PVA/GO–AgNPs composite films can be potential materials in the biomedical field as wound dressings for wound healing and infection prevention.

## 4. Conclusions

GO–AgNPs filled PVA composite films with antibacterial activity were prepared by solution casting method. Microstructural and morphological characterization revealed the good dispersion of the filler in the polymer matrix. The exfoliated structure of the nanocomposites resulted in enhanced thermal stability, mechanical properties and water resistance. PVA films filled with 0.5 to 5 wt % GO–AgNPs showed antibacterial effect against *E. coli* and *S. aureus*, being stronger for the composite with higher GO–AgNPs content. PVA/GO–AgNPs films caused higher inactivation of *S. aureus* compared to *E. coli*. The direct contact of the bacteria with the composite films led a higher bactericidal effect than the contact with the leachates from those films. PVA/GO–AgNPs can be regarded as promising materials for potential applications in the biomedical field.

## Figures and Tables

**Table 1:** Reaction conditions for the synthesis of GO–AgNPs and average size of AgNPs formed.

Reaction Conditions		AgNPs
AgNO_3_ Concentration (mM)	Temperature (°C)	Diameter (nm)
1.50	60	3.1 ± 0.8

**Table 2:** Differential scanning calorimetry (DSC) results of neat PVA and PVA/GO–AgNPs nanocomposites.

Sample	*T_g_* (°C)	*T_m_* (°C)	Δ*H_m_* (J/g)	*T_c_* (°C)	Δ*H_c_* (J/g)	*X_c_* (%)
PVA	76.5	221.7	73.6	197.7	62.5	51.9
PVA/GO–AgNPs0.5	78.3	222.8	70.0	201.8	57.9	49.6
PVA/GO–AgNPs1	78.6	223.8	61.5	201.8	50.4	43.8
PVA/GO–AgNPs2	79.6	224.6	61.8	202.5	53.5	44.4
PVA/GO–AgNPs5	83.0	223.5	59.3	202.8	48.2	44.0

**Table 3:** TGA data for neat PVA and PVA/GO–AgNPs nanocomposites.

Sample	*T_5_* (°C)	*T_50_* (°C)	*T_max_* (°C)			Residue (%)
			(a)	(b)	(c)	
PVA	271	338	302	350	425	2.9
PVA/GO–AgNPs0.5	274	343	308	355	428	3.2
PVA/GO–AgNPs1	273	341	308	353	428	3.8
PVA/GO–AgNPs2	280	353	314	357	429	4.9
PVA/GO–AgNPs5	285	354	312	356	431	7.4

## Supplementary Materials

The following are available online at https://www.mdpi.com/2073-4360/12/3/723/s1; Preparation of Graphene Oxide (GO). Physicochemical Characterization. Figure S1: (A) FTIR, (B) UV-vis and (C) XRD spectra of GO and GO–AgNPs hybrid. Figure S2: C1s XPS spectrum of (A) GO and (B) GO–AgNPs; (C) Ag3d spectrum of GO–AgNPs; (D) Raman spectra of GO and GO–AgNPs. Figure S3: (A) TEM image of GO–AgNPs hybrid, inset shows SAED pattern; (B) AgNPs size distribution. Figure S4: TGA curves of GO and GO–AgNPs hybrid. Figure S5: UV-vis absorption spectra of leachates from PVA/GO–AgNPs nanocomposite films after water immersion.

## References

[1] Hong K.H., Park J.L., Sul I.H., Youk J.H., Kang T.J. (2006). Preparation of antimicrobial poly(vinyl alcohol) nanofibers containing silver nanoparticles. J. Polym. Sci. Part B Polym. Phys..

[2] Li Q., Mahendra S., Lyon D.Y., Brunet L., Liga M.V., Li D., Alvarez P.J.J. (2008). Antimicrobial nanomaterials for water disinfection and microbial control: Potential applications and implications. Water Res..

[3] Venkatesham M., Ayodhya D., Madhusudhan A., Veera Babu N., Veerabhadram G. (2012). A novel green one-step synthesis of silver nanoparticles using chitosan: Catalytic activity and antimicrobial studies. Appl. Nanosci..

[4] Akhavan O., Ghaderi E. (2010). Toxicity of graphene and graphene oxide nanowalls against bacteria. ACS Nano.

[5] Liu S., Zeng T.H., Hofmann M., Burcombe E., Wei J., Jiang R., Kong J., Chen Y. (2011). Antibacterial Activity of Graphite, Graphite Oxide, Graphene Oxide, and Reduced Graphene Oxide: Membrane and Oxidative Stress. ACS Nano.

[6] Liu S., Hu M., Zeng T.H., Wu R., Jiang R., Wei J., Wang L., Kong J., Chen Y. (2012). Lateral dimension-dependent antibacterial activity of graphene oxide sheets. Langmuir.

[7] Akhavan O., Ghaderi E., Esfandiar A. (2011). Wrapping bacteria by graphene nanosheets for isolation from environment, reactivation by sonication, and inactivation by near-infrared irradiation. J. Phys. Chem. B.

[8] Hu W., Peng C., Luo W., Lv M., Li X., Li D., Huang Q., Fan C. (2010). Graphene-Based Antibacterial Paper. ACS Nano.

[9] Kurantowicz N., Sawosz E., Jaworski S., Kutwin M., Strojny B., Wierzbicki M., Szeliga J., Hotowy A., Lipińska L., Kozinski R. (2015). Interaction of graphene family materials with *Listeria monocytogenes* and *Salmonella enterica*. Nanoscale Res. Lett..

[10] Chen J., Peng H., Wang X., Shao F., Yuan Z., Han H. (2014). Graphene oxide exhibits broad-spectrum antimicrobial activity against bacterial phytopathogens and fungal conidia by intertwining and membrane perturbation. Nanoscale.

[11] Raza M.A., Kanwal Z., Rauf A., Sabri A.N., Riaz S., Naseem S. (2016). Size- and shape-dependent antibacterial studies of silver nanoparticles synthesized by wet chemical routes. Nanomaterials.

[12] Gurunathan S. (2014). Rapid biological synthesis of silver nanoparticles and their enhanced antibacterial effects against *Escherichia fergusonii* and *Streptococcus mutans*. Arab. J. Chem..

[13] Jeong Y., Lim D.W., Choi J. (2014). Assessment of size-dependent antimicrobial and cytotoxic properties of silver nanoparticles. Adv. Mater. Sci. Eng..

[14] Carlson C., Hussain S.M., Schrand A.M., Braydich-Stolle L.K., Hess K.L., Jones R.L., Schlager J.J. (2008). Unique cellular interaction of silver nanoparticles: Size-dependent generation of reactive oxygen species. J. Phys. Chem. B.

[15] Guzmán M.G., Dille J., Godet S. (2009). Synthesis of silver nanoparticles by chemical reduction method and their antibacterial activity. Int. J. Chem. Biomol. Eng..

[16] Zhu Z., Su M., Ma L., Ma L., Liu D., Wang Z. (2013). Preparation of graphene oxide–silver nanoparticle nanohybrids with highly antibacterial capability. Talanta.

[17] Tang J., Chen Q., Xu L., Zhang S., Feng L., Cheng L., Xu H., Liu Z., Peng R. (2013). Graphene oxide−silver nanocomposite as a highly effective antibacterial agent with species-specific mechanisms. ACS Appl. Mater. Interface.

[18] Santos C.M., Mangadlao J., Ahmed F., Leon A., Advincula R.C., Rodrigues D.F. (2012). Graphene nanocomposite for biomedical applications: Fabrication, antimicrobial and cytotoxic investigations. Nanotechnology.

[19] Damm C., Münstedt H., Rösch A. (2007). Long-term antimicrobial polyamide 6/silver-nanocomposites. J. Mater. Sci..

[20] Tamayo L.A., Zapata P.A., Vejar N.D., Azocar M.I., Gulppi M.A., Zhou X., Thompson G.E., Rabagliati F.M., Paez M.A. (2014). Release of silver and copper nanoparticles from polyethylene nanocomposites and their penetration into *Listeria monocytogenes*. Mater. Sci. Eng. C Mater. Biol. Appl..

[21] Zapata P.A., Tamayo L., Páez M., Cerda E., Azócar I., Rabagliati F.M. (2011). Nanocomposites based on polyethylene and nanosilver particles produced by metallocenic “in situ” polymerization: Synthesis, characterization, and antimicrobial behavior. Eur. Polym. J..

[22] Arriagada P., Palza H., Palma P., Flores M., Caviedes P. (2018). Poly(lactic acid) composites based on graphene oxide particles with antibacterial behavior enhanced by electrical stimulus and biocompatibility. J. Biomed. Mater. Res. A.

[23] Lim H.N., Huang N.M., Loo C.H. (2012). Facile preparation of graphene-based chitosan films: Enhanced thermal, mechanical and antibacterial properties. J. Non-Cryst. Solids.

[24] Carpio I.E.M., Santos C.M., Wei X., Rodrigues D.F. (2012). Toxicity of a polymer—Graphene oxide composite against bacterial planktonic cells, biofilms, and mammalian cells. Nanoscale.

[25] Cobos M., De-La-Pinta I., Quindós G., Fernández M.J., Fernández M.D. (2019). One-step eco-friendly synthesized silver-graphene oxide/poly(vinyl alcohol) antibacterial nanocomposites. Carbon.

[26] Surudžić R., Janković A., Bibić N., Vukašinović-Sekulić M., Perić-Grujić A., Mišković-Stanković V., Park S.J., Rhee K.Y. (2016). Physico-chemical and mechanical properties and antibacterial activity of silver/poly(vinyl alcohol)/graphene nanocomposites obtained by electrochemical method. Compos. Part B Eng..

[27] Usman A., Hussain Z., Riaz A., Khan A.N. (2016). Enhanced mechanical, thermal and antimicrobial properties of poly(vinyl alcohol)/graphene oxide/starch/silver nanocomposites films. Carbohydr. Polym..

[28] Ma Y., Bai D., Hu X., Ren N., Gao W., Chen S., Chen H., Lu Y., Li J., Bai Y. (2018). Robust and antibacterial polymer/mechanically exfoliated graphene nanocomposite fibers for biomedical applications. ACS Appl. Mater. Interfaces.

[29] Liu C., Shen J., Yeung K.W.K., Tjong S.C. (2017). Development and antibacterial performance of novel polylactic acid-graphene oxide-silver nanoparticle hybrid nanocomposite mats prepared by electrospinning. ACS Biomater. Sci. Eng..

[30] Bhunia S.K., Jana N.R. (2014). Reduced graphene oxide-silver nanoparticle composite as visible light photocatalyst for degradation of colorless endocrine disruptors. ACS Appl. Mater. Interfaces.

[31] He K., Zeng Z., Chen A., Zeng G., Xiao R., Xu P., Huang Z., Shi J., Hu L., Chen G. (2018). Advancement of Ag–graphene based nanocomposites: An overview of synthesis and its applications. Small.

[32] Yang R., Dong F., You X., Liu M., Shan Z., Lishan Z., Lishan B. (2019). Facile synthesis and characterization of interface charge transfer heterojunction of Bi2MoO6 modified by Ag/AgCl photosensitive material with enhanced photocatalytic activity. Mater. Lett..

[33] Liu Y., Hou C., Jiao T., Song J., Zhang X., Xing R., Zhou J., Zhang L., Peng Q. (2018). Self-assembled AgNP-containing nanocomposites constructed by electrospinning as efficient dye photocatalyst materials for wastewater treatment. Nanomaterials.

[34] Ding K., Wang W., Yu D., Wang W., Gao P., Liu B. (2018). Facile formation of flexible Ag/AgCl/polydopamine/cotton fabric composite photocatalysts as an efficient visible-light photocatalysts. Appl. Surf. Sci..

[35] Liang J., Huang Y., Zhang L., Wang Y., Ma Y., Guo T., Chen Y. (2009). Molecular-level dispersion of graphene into poly(vinyl alcohol) and effective reinforcement of their nanocomposites. Adv. Funct. Mater..

[36] Zhao X., Zhang Q., Chen D., Lu P. (2010). Enhanced mechanical properties of graphene-based poly(vinyl alcohol) composites. Macromolecules.

[37] Kashyap S., Pratihar S.K., Behera S.K. (2016). Strong and ductile graphene oxide reinforced PVA nanocomposites. J. Alloy. Compd..

[38] Yang X., Shang S., Li L. (2011). Layer-structured poly(vinyl alcohol)/graphene oxide nanocomposites with improved thermal and mechanical properties. J. Appl. Polym. Sci..

[39] Bao C., Guo Y., Song L., Hu Y. (2011). Poly(vinyl alcohol) nanocomposites based on graphene and graphite oxide: A comparative investigation of property and mechanism. J. Mater. Chem..

[40] Liu D., Bian Q., Li Y., Wang Y., Xiang A., Tian H. (2016). Effect of oxidation degrees of graphene oxide on the structure and properties of poly (vinyl alcohol) composite films. Compos. Sci. Technol..

[41] Cobos M., González B., Fernández M.J., Fernández M.D. (2017). Chitosan–graphene oxide nanocomposites: Effect of graphene oxide nanosheets and glycerol plasticizer on thermal and mechanical properties. J. Appl. Polym. Sci..

[42] Cobos M., De-La-Pinta I., Quindós G., Fernández M.J., Fernández M.D. (2020). Graphene oxide–silver nanoparticle nanohybrids: Synthesis, characterization, and antimicrobial properties. Nanomaterials.

[43] Antimicrobial Susceptibility Testing. http://www.eucast.org/ast_of_bacteria/.

[44] Nishio Y., Haratani T., Takahashi T., Manley R.S.J. (1989). Cellulose/Poly(vinyl alcohol) blends: An estimation of thermodynamic polymer-polymer interaction by melting point depression analysis. Macromolecules.

[45] Tsuchiya Y., Sumi K. (1969). Thermal decomposition products of poly(viny1 alcohol). J. Polym. Sci. Pol. Chem..

[46] Wang J., Yang S., Huang Y., Tien H., Chin W., Ma C.M. (2011). Preparation and properties of graphene oxide/polyimide composite films with low dielectric constant and ultrahigh strength via in situ polymerization. J. Mater. Chem..

[47] Kim H., Abdala A.A., Macosko C.W. (2010). Graphene/polymer nanocomposites. Macromolecules.

[48] Suk J.W., Piner R.D., An J., Ruoff R.S. (2010). Mechanical properties of monolayer graphene oxide. ACS Nano.

[49] Yang X., Li L., Shang S., Tao X. (2010). Synthesis and characterization of layer-aligned poly(vinylalcohol)/graphene nanocomposites. Polymer.

[50] Manna S., Batabyal S.K., Nandi A.K. (2006). Preparation and characterization of silver-poly(vinylidene fluoride) nanocomposites: Formation of piezoelectric polymorph of poly(vinylidene fluoride). J. Phys. Chem. B.

[51] Mbhele Z.H., Salemane M.G., van Sittert C.G.C.E., Nedeljkovic J.M., Djokovic V., Luyt A.S. (2003). Fabrication and characterization of silver-polyvinyl alcohol nanocomposites. Chem. Mater..

[52] Wang J., Wang X., Xu C., Zhang M., Shang X. (2011). Preparation of graphene/poly(vinyl alcohol) nanocomposites with enhanced mechanical properties and water resistance. Polym. Int..

[53] Cobos M., Fernández M.J., Fernández M.D. (2018). Graphene based poly(viny lalcohol) nanocomposites prepared by in situ green reduction of graphene oxide by ascorbic acid: Influence of graphene content and glycerol plasticizer on properties. Nanomaterials.

[54] Adame D., Beall G.W. (2009). Direct measurement of the constrained polymer region in polyamide/clay nanocomposites and the implications for gas diffusion. Appl. Clay Sci..

[55] Rao Y., Pochan J.M. (2007). Mechanics of polymer-clay nanocomposites. Macromolecules.

[56] Gurunathan S., Han J.W., Dayem A.A., Eppakayala V., Kim J.-H. (2012). Oxidative stress-mediated antibacterial activity of graphene oxide and reduced graphene oxide in Pseudomonas aeruginosa. Int. J. Nanomed..

[57] Perreault F., Fonseca de Faria A., Nejati S., Elimelech M. (2015). Antimicrobial properties of graphene oxide nanosheets: Why size matters. ACS Nano.

[58] Morones J.R., Elechiguerra J.L., Camacho A., Holt K., Kouri J.B., Ramirez J.T., Yacaman M.J. (2005). The bactericidal effect of silver nanoparticles. Nanotechnology.

[59] Kim S., Choi J.E., Choi J., Chung K.H., Park K., Yi J., Ryu D.Y. (2009). Oxidative stress dependent toxicity of silver nanoparticles in human hepatoma cells. Toxicol. Vitro.

[60] Xiu Z., Zhang Q., Puppala H.L., Colvin V.L., Alvarez P.J.J. (2012). Negligible particle-specific antibacterial activity of silver nanoparticles. Nano Lett..

[61] Bondarenko O., Ivask A., Käkinen A., Kurvet I., Kahru A. (2013). Particle-cell contact enhances antibacterial activity of silver nanoparticles. PLoS ONE.

[62] Gunawan C., Teoh W.Y., Marquis C.P., Lifia J., Amal R. (2009). Reversible antimicrobial photoswitching in nanosilver. Small.

[63] Wigginton N.S., Titta A., Piccapietra F., Dobias J., Nesatyy V.J., Suter M.J.F., Bernier-Latmani R. (2010). Binding of silver nanoparticles to bacterial proteins depends on surface modifications and inhibits enzymatic activity. Environ. Sci. Technol..

[64] Navarro E., Piccapietra F., Wagner B., Marconi F., Kaegi R., Odzak N., Sigg L., Behra R. (2008). Toxicity of silver nanoparticles to Chlamydomonas reinhardtii. Environ. Sci. Technol..

[65] Kawata K., Osawa M., Okabe S. (2009). In vitro toxicity of silver nanoparticles at noncytotoxic doses to HepG2 human hepatoma cells. Environ. Sci. Technol..

[66] Beer C., Foldbjerg R., Hayashi Y., Sutherland D.S., Autrup H. (2012). Toxicity of silver nanoparticles—Nanoparticle or silver ion?. Toxicol. Lett..

[67] Samberg M.E., Orndorff P.E., Monteiro-Riviere N.A. (2011). Antibacterial efficacy of silver nanoparticles of different sizes, surface conditions and synthesis methods. Nanotoxicology.

[68] Sondi I., Salopek-Sondi B. (2004). Silver nanoparticles as antimicrobial agent: A case study on E. coli as a model for Gram-negative bacteria. J. Colloid Interface Sci..

[69] Agnihotri S., Mukherji S., Mukherji S. (2014). Size-controlled silver nanoparticles synthesized over the range 5-100 nm using the same protocol and their antibacterial efficacy. RSC Adv..

[70] Sotiriou G.A., Pratsinis S.E. (2010). Antibacterial activity of nanosilver ions and particles. Environ. Sci. Technol..

[71] Zhao H., Ding R., Zhao X., Li Y., Qu L., Pei H., Yildirimer L., Wu Z., Zhang W. (2017). Graphene-based nanomaterials for drug and/or gene delivery, bioimaging, and tissue engineering. Drug Discov. Today.

[72] Zhang T., Wang L., Chen Q., Chen C. (2014). Cytotoxic potential of silver nanoparticles. Yonsei Med. J..

[73] Plachá D., Jampilek J. (2019). Graphenic materials for biomedical applications. Nanomaterials.

[74] Li L., Sun J., Li X., Zhang Y., Wang Z., Wang C., Dai J., Wang Q. (2012). Controllable synthesis of monodispersed silver nanoparticles as standards for quantitative assessment of their cytotoxicity. Biomaterials.

[75] Park M.V., Neigh A.M., Vermeulen J.P., de la Fonteyne L.J., Verharen H.W., Briedé J.J., van Loveren H., de Jong W.H. (2011). The effect of particle size on the cytotoxicity, inflammation, developmental toxicity and genotoxicity of silver nanoparticles. Biomaterials.

[76] Sowa-Söhle E.N., Schwenke A., Wagener P., Weiss A., Wiegel H., Sajti C.L., Haverich A., Barcikowski S., Loos A. (2013). Antimicrobial efficacy, cytotoxicity and ion release of mixed metal (Ag, Cu, Zn, Mg) nanoparticle polymer composite implant material. BioNanoMaterials.

[77] Oliveira R.N., Rouze R., Quilty B., Alves G.G., Soares G.D.A., Thire R.M., McGuinness G.B. (2014). Mechanical properties and in vitro characterization of polyvinyl alcohol-nano-silver hydrogel wound dressings. Interface Focus.

[78] Bosetti M., Masse A., Tobin E., Cannas M. (2002). Silver coated materials for external fixation devices: In vitro biocompatibility and genotoxicity. Biomaterials.

[79] Paladini F., Pollini M., Sannino A., Ambrosio L. (2015). Metal-based antibacterial substrates for biomedical applications. Biomacromolecules.

[80] Li Y.Q., Yu T., Yang T.Y., Zheng L.X., Liao K. (2012). Bio-inspired nacre-like composite films based on graphene with superior mechanical, electrical, and biocompatible properties. Adv. Mater..

