# Supplementary material for: Synthesis, Physical, Mechanical and Antibacterial Properties of Nanocomposites Based on Poly(vinyl alcohol)/Graphene Oxide–Silver Nanoparticles
Source: Polymers (Basel). 2020 Mar 24;12(3):723. doi: 10.3390/polym12030723 (PMC7183062; doi:10.3390/polym12030723)
Supplement: Supplementary file 1 [file polymers-12-00723-s001.pdf]

polymers

*Supplementary Materials*

# Synthesis, Physical, Mechanical and Antibacterial Properties of Nanocomposites based on Poly(vinyl alcohol)/Graphene Oxide–Silver Nanoparticles

**Mónica Cobos [1], Iker De-La-Pinta [2], Guillermo Quindós [2], M. Jesús Fernández [1], M. Dolores Fernández [1,*]**

[1] Department of Polymer Science and Technology, Faculty of Chemistry, University of the Basque Country (UPV/EHU), Paseo Manuel Lardizábal 3, 20018 San Sebastián, Spain; monica.cobos@ehu.es (M.C.); mjesus.fernandez@ehu.es (M.J.F.)
[2] Department of Immunology, Microbiology and Parasitology, Faculty of Medicine and Nursing, University of the Basque Country (UPV/EHU), Barrio Sarriena s/n, Leioa 48940, Spain; iker.delapinta@ehu.es (I.D.L.P.); guillermo.quindos@ehu.eus (G.Q.)
* Correspondence: mariadolores.fernandez@ehu.es (M.D.F.); Tel.: +34-943-01-8179

## *Preparation of Graphene Oxide (GO)*

A flask containing a mixture of 2 g graphite, 50 mL $H_2SO_4$ and 1 g $NaNO_3$ was placed in a water bath at 0 °C. Then, 6 g of $KMnO_4$ was slowly added, and the mixture was subsequently heated up to 35 °C for 1 h. After the slow addition of 100 mL of de-ionized water, 10 mL of 30% $H_2O_2$ was incorporated into the solution. The suspension was subjected to centrifugation to separate the solid, that was washed with water, HCl and finally again with water until reaching neutral pH. Finally, the product, graphite oxide (GrO), was obtained by freeze-drying. GO was obtained after ultrasonication of an aqueous dispersion of GrO.

## *Physicochemical Characterization*

### Structure and Morphology

FTIR spectra were recorded using a Thermo Nicolet iS10 spectrometer (Thermo Fisher Scientific, Madison, WI, USA) equipped with an attenuated total reflectance accessory (ATR). UV-Vis spectra were measured on a Perkin Elmer Lambda 25 spectrometer (Shelton, CT, USA) in the range 200-800 nm. Surface chemistry characterization of graphenic materials was performed by XPS on a SPECS system with a monochromatic Al Kα X-ray source (1486.6 eV) and a Phoibos 150 1D-DLD analyzer (Berlin, Germany). The core level spectra were obtained at a photoelectron take-off angle of 90°, measured with respect to the sample surface. The XPS survey-scan spectra were taken with pass energy of 80 eV, 1 eV energy step, and 0.1 s dwell time. The individual high-resolution spectra were acquired with pass energy of 30 eV, 0.1 eV energy step, and 0.1 s dwell time. Raman characterization of GO and GO–AgNPs were carried out with a Renishaw Invia microscope (Gloucestershire, UK) with laser frequency of 514 nm as an excitation source. The spectra were measured from 500 to 3500 $cm^{-1}$. XRD spectra of graphenic materials, neat PVA and PVA/GO–AgNPs nanocomposites were performed using a Malvern Panalytical (Almelo, Netherlands) X'PERT PRO automatic diffractometer operating at 40 kV and 40 mA, in theta-theta configuration, secondary monochromator with Cu-Kα radiation ($\lambda$ = 1.5418 Å) and a PIXcel solid state detector (active length in 2$\theta$ 3.347°). Data were collected from 1 to 50° 2$\theta$ for GO and PVA, and from 1 to 90° 2$\theta$ for GO–AgNPs and PVA/GO–AgNPs

samples (step size = 0.026° and time per step = 80 s, total time 20 min) at room temperature. A variable divergence slit giving a constant 5mm area of sample illumination was used. The interlayer separation in the graphenic material was calculated by the Bragg´s law ($\lambda$ = 2d sin$\theta$). TEM measurements were carried out using a Philips Tecnai G2 20 TWIN TEM instrument (Eindhoven, Netherlands) at 200 kV accelerated voltage. GO–AgNPs samples were prepared by drop-coating dilute suspensions onto carbon coated copper grids and dried at room temperature. PVA/GO–AgNPs nanocomposites were sectioned with a Leica EM UC6 ultramicrotome apparatus and placed in coated cooper grids. SEM images were also obtained for PVA and PVA/GO–AgNPs films, using a Hitachi S-4800 scanning electron microscope (Tokyo, Japan) operated at 5 kV accelerating voltage. Samples were fractured in liquid nitrogen and the cross-sections were sputtered with gold, after which the cross-section morphology was examined.

***Thermal characterization***

Thermogravimetric analysis (TGA) and differential scanning calorimetry analyses (DSC) were used for thermal characterization. TGA tests were performed on a TG-Q-500 (TA instruments, New Castle, DE, USA) under nitrogen at a heating rate of 10 °C/min, from 40 to 800 °C. DSC thermograms were recorded with a a Mettler Toledo DSC 3+ unit (Greifensee, Switzerland), at a heating rate of 10°C/min under nitrogen atmosphere at a flow rate of 20 mL/min, over a temperature range of -30 to 250 °C.

***Mechanical characterization***

The mechanical characterization was conducted in an electromechanical testing machine (Instron 5967, Norwood, MA, USA) with a 500 N load cell, a displacement rate of 5 mm/min, and a gauge length of 10 mm. Dog-bone shaped specimens were punched out of the films, and maintained at room temperature at a relative humidity of 58 % for one week prior to testing, to guarantee moisture equilibrium of the specimens. Ten specimens of each sample were tested.

***Characterization of GO and GO–AgNPs hybrid***

The successful synthesis of graphene oxide (GO) and GO–AgNPs hybrid was confirmed by FTIR, UV-vis, X-ray diffraction, XPS and Raman spectroscopy. Figure S1 compares FTIR, UV-vis and XRD spectra of GO and GO–AgNPs hybrid. FTIR spectrum of GO shows the strong bands related to the C=O carbonyl stretching vibrations of the COOH groups at 1734 $cm^{-1}$, located at the edges of GO sheets, the bending of tertiary C-OH groups at 1362 $cm^{-1}$, and the stretching vibration of C-O of epoxide groups at 1052 $cm^{-1}$ (Figure S1A). The strong band at 3800-300 $cm^{-1}$ is assigned to the stretching vibrations of structural OH groups and physisorbed water molecules. Regarding the FTIR spectrum of GO–AgNPs hybrid, the weakening of the mentioned bands can be observed, suggesting the simultaneous partial reduction of GO.

The comparison of the UV-vis spectra of GO and GO–AgNPs shows the red shift of the GO peak from 230 to 265 nm, the disappearance of the shoulder at 303 nm, and the appearance of the surface plasmon resonance band of AgNPs at 404 nm, when AgNPs are incorporated on the surface of GO (Figure S1B). These results confirm the partial reduction of GO and the presence of small spherical shape AgNPs attached to GO surface.

XRD patterns of GO and GO–AgNPs are presented in Figure S1C. The XRD pattern of GO exhibits 2$\theta$ peak at 11.1°, whereas the hybrid displays peaks from 2$\theta$ = 30° to 90°, but the peak of GO is not seen. Those peaks correspond to the (111), (200), (311) and (222) planes of the face centred cubic of AgNPs. All these results indicated the decoration of GO with AgNPs and the exfoliation of GO–AgNPs sheets.

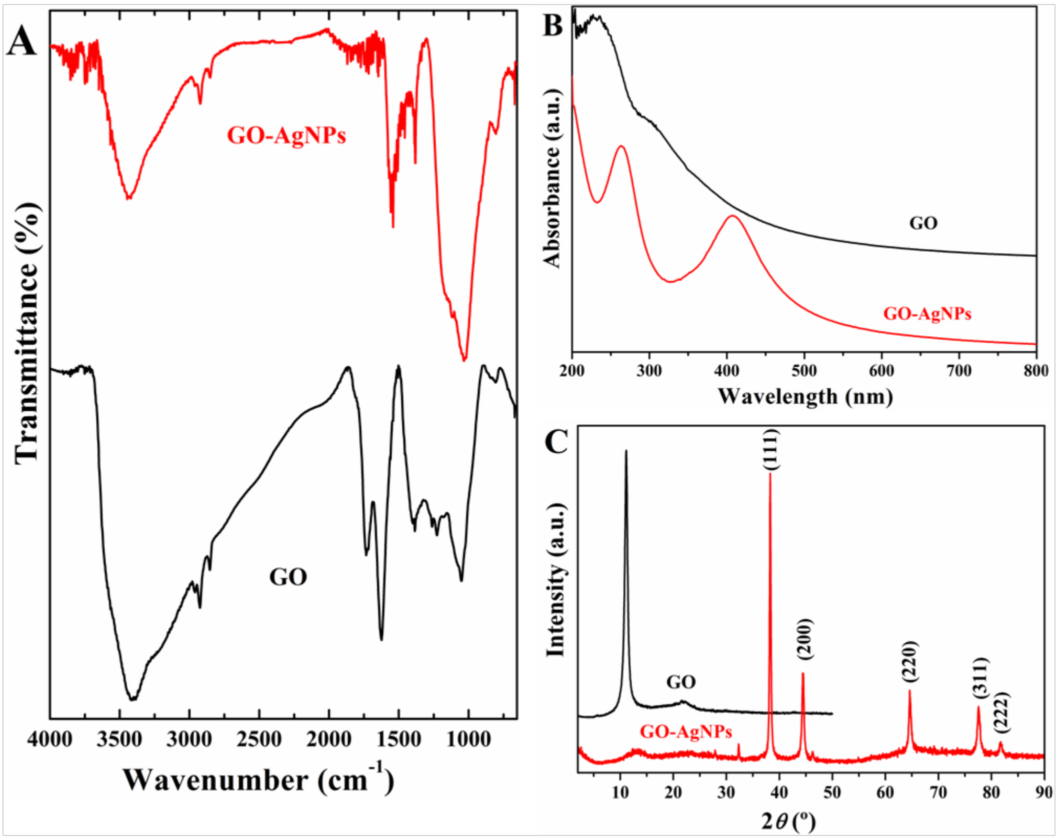


**Figure S1**. (**A**) FTIR, (**B**) UV-vis and (**C**) XRD spectra of GO and GO–AgNPs hybrid.

The C1s XPS spectra for GO and GO–AgNPs are shown in Figures S2A and B, respectively. The intensity of C-O peak is significantly reduced, the C-C peak narrows and the shake-up satellite at ~290 eV appears after the formation of AgNPs on the GO surface. These results confirm the simultaneous partial reduction of GO during the preparation of the hybrid. The presence of Ag3d5/2 and Ag3d3/2 peaks found at 368.4 and 374.4 eV, respectively, in the Ag3d spectrum (Figure S2C) indicate the formation of metallic silver.

Raman spectra of GO and GO–AgNPs hybrid is provided in Figure 2D. There is a shift of all the bands to lower wavenumbers, an increase of the intensity of D band and an increase of the intensity ratio $I_D/I_G$ after the attachment of AgNPs to the GO surface. From these results, it can be inferred that structural defects have been introduced in the graphene sheets.

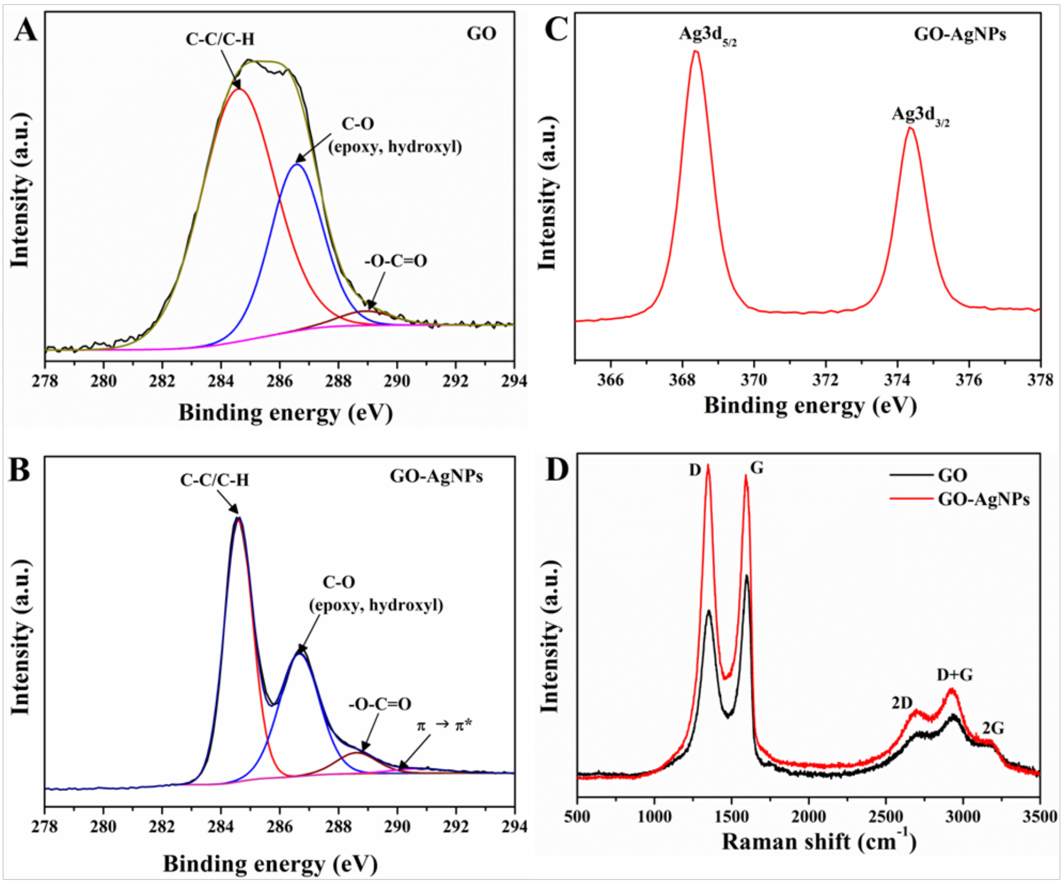


**Figure S2**. C1s XPS spectrum of (**A**) GO and (**B**) GO–AgNPs; (C) Ag3d spectrum of GO–AgNPs; (D) Raman spectra of GO and GO–AgNPs.

The morphology and structure of the GO–AgNPs hybrid was analysed by TEM. The image of Figure S3A shows the spherical AgNPs, with average size of 3.1±0.8 nm, distributed uniformly over the surface of GO. AgNPs size was determined by measuring the diameter of 1200 nanoparticles in several TEM images. The histogram shows a narrow size distribution, with most of particles under 4 nm in diameter (Figure S3B). The selected area electron diffraction (SAED) pattern obtained from the hybrid is shown as an inset in Figure S3A. It shows the diffraction rings corresponding to (111), (200), (220) and (311) of Ag, indicating the polycrystalline nature of silver.

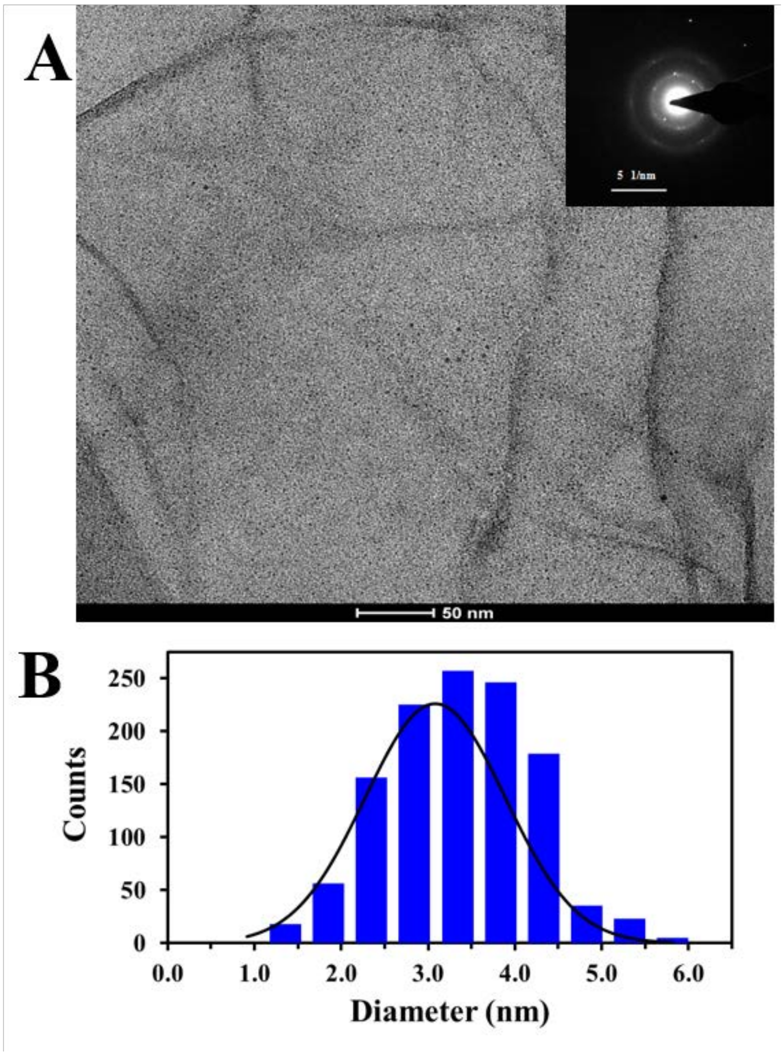


**Figure S3**. (**A**) TEM image of GO–AgNPs hybrid, inset shows SAED pattern. (**B**) AgNPs size distribution.

***Thermal stability of GO and GO–AgNPs hybrid***

Thermogravimetric analysis (TGA) was used to evaluate the thermal stability of the graphenic materials. Figure S4 shows the TGA mass loss curves. Both curves show two decomposition steps, the first one is located below 120 °C and the second one is found in the range 140-300 °C. The first weight loss is attributed to the elimination of the interlaminar water, whereas in the second step less stable oxygen containing functional groups decompose. The mass loss during this step was about 32% and 16% for GO and GO–AgNPs hybrid, respectively. The lower mass loss for the hybrid indicates that GO was partially reduced during the synthesis process of the hybrid.

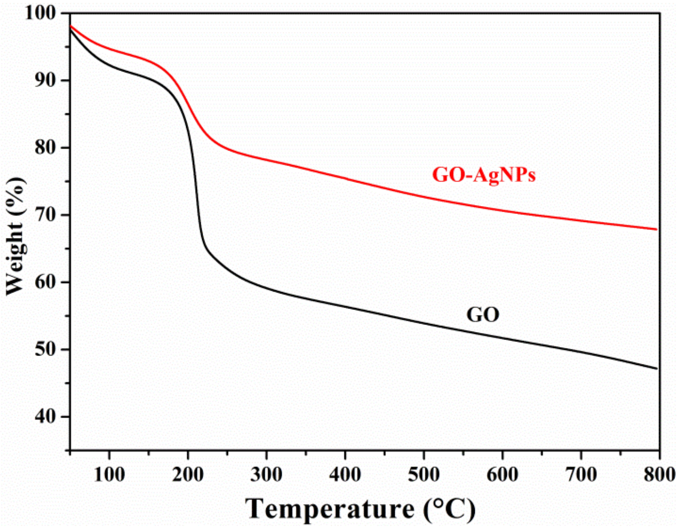


**Figure S4**. TGA curves of GO and GO–AgNPs hybrid.

***Lixiviation test***

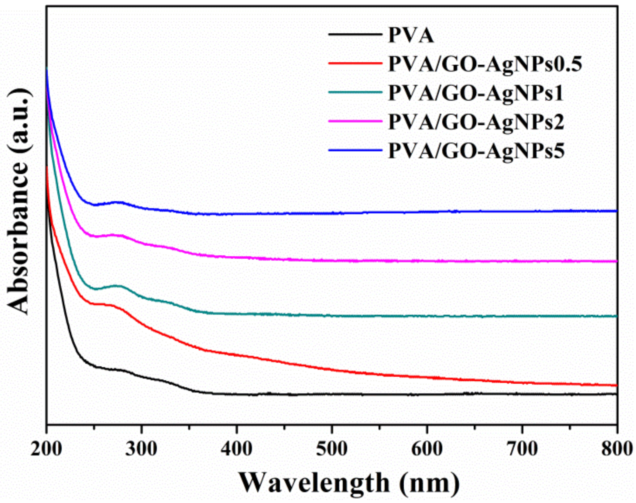


**Figure S5**. UV-vis absorption spectra of leachates from PVA/GO–AgNPs nanocomposite films after water immersion.
